# “*Are they going to recollect who they need to contact?*”: understanding sexually transmitted infection transmission risks among older Canadians who winter in the United States

**DOI:** 10.1186/s40794-025-00245-4

**Published:** 2025-05-07

**Authors:** Olivia Nieves Echevarria, John Pickering, Valorie A. Crooks, Jeremy Snyder, Trudie Milner

**Affiliations:** 1 Department of Geography, Simon Fraser University, Burnaby, BC Canada; 2 Gerontology Research Centre, Simon Fraser University, Burnaby, BC Canada; 3 Faculty of Health Sciences, Simon Fraser University, Burnaby, BC Canada; 4 Yuma Regional Medical Center, Yuma, AZ United States

**Keywords:** International retirement migration, Canada, United States, Sexually transmitted infections, Sexual health risks, Stigma

## Abstract

**Background:**

Sexually transmitted infections are on the rise in older populations globally, including among older travellers. International retirement migrants are older people who have retired from the workforce and travel abroad seasonally, typically during the winter months in their home countries. The transnational nature of this practice may challenge public health efforts to control the spread of sexually transmitted infection and encourage treatment. This study focuses on Yuma, Arizona, a popular destination for Canadian international retirement migrants who winter in the United States, to examine the sexual health risks associated with their seasonal travel.

**Methods:**

Utilizing a qualitative case study approach, this research involved semi-structured interviews conducted remotely with key informants in Yuma (*n* = 10) who held various health care and administrative roles. Participants provided insights into sexual health risks based on their extensive interactions with Canadian seasonal migrants and their knowledge of the social dynamics within retirement communities. Interviews were transcribed verbatim, coded using NVivo software, and thematically analyzed to identify risk factors for sexually transmitted infections among Canadian international retirement migrants wintering in Yuma.

**Results:**

Findings revealed three main risks that may contribute to exposure to sexually transmitted infections and potential transmission: social dynamics within tight-knit retirement migrant communities that facilitate unsafe sexual practices (i.e., risky practices); barriers to accessing diagnostic services, such as costs and lack of established local care (i.e., risky care access); and challenges in following standard treatment and public health protocols due to logistical difficulties in ensuring follow-up (i.e., risky treatment decisions). Key informants noted that lifestyle choices, including the use of alcohol and drugs, can exacerbate these risks. Health care access barriers driven by travel health insurance and mobility limitations further complicate the diagnosis and treatment of sexually transmitted infections for Canadian international retirement migrants while abroad.

**Conclusions:**

This study highlights the complex interplay of social behaviours and health care barriers that heighten the risk of sexually transmitted infection transmission among Canadian retirement migrants in the transnational context of Yuma. Extended diagnostic and treatment services, comprehensive sexual health education in pre- and post-travel consultations, as well as inclusive travel health insurance coverage could significantly improve the sexual health outcomes for this population.

## Introduction

International travel has been linked to the spread of sexually transmitted infections (STIs) [1, 2], even though their early asymptomatic nature and incubation time cause difficulties in establishing transmission location [2]. Travel, for example, can foster a sense of anonymity that may lead to altered sexual behaviour and increased exposure to STIs [1–3]. Drug and alcohol consumption also increases during some travel, which are significant risk factors for acquiring STIs due to unsafe or casual sexual practices [4, 5]. Despite these risks, sexual health is seldom addressed in pre-travel health consultations due to various barriers, including time constraints, limited resources, and prevailing social norms that deem sexual behaviours to be a private matter [6]. However, it is imperative to better understand the increased sexual risks faced by travelers, particularly those who frequently travel or stay abroad for extended periods, given the implications for public health and health systems. Longer stay travellers may especially underestimate their vulnerability to exposure due to familiarity with the destination, leading to more opportunities for engaging in risky sexual encounters [4].

International retirement migrants, typically people around the age of retirement and beyond, engage in seasonal relocations, usually from colder to warmer climates and during the winter months [7]. These migrations can range from seasonal stays to permanent relocations [8] and may involve second-home ownership [9]. Our focus here is on Canadian international retirement migrants who travel seasonally to the United States (US) for weeks to months of Canada’s winter. This group, largely comprising affluent ‘baby boomers’ [10], is drawn to destinations that offer warm weather, cultural and natural attractions, robust social networks, and affordable living [11]. These seasonal travellers are untracked, making precise numbers impossible to determine. However, recent estimates suggest 500,000 to more than one million older Canadians winter annually in the US [12]. Those whose health declines cease their annual travel [13, 14], resulting in seasonal expatriate communities throughout the southern US composed predominantly of healthy, active seniors. This vitality may extend to continued sexual activity, as barriers to sexual engagement are generally not prohibitive until health decline is severe [15]. Understanding how sexual health is impacted by the lifestyle of international retirement migrants is critical, particularly given the global rise in STIs among older adults and the significant numbers of older people who travel abroad seasonally [16, 17].

In the US, the rise in STIs among older people has been widely documented [18]. The majority of Canadian international retirement migrants congregate in a few US states – Florida, Arizona, California, Hawaii and Texas – where they spend the weeks or months between November and April [17]. Many older Canadians avoid using seemingly costly health care while in the US, opting instead for ‘stocking up’ strategies that include making pre-trip physician visits and filling prescriptions pre-travel [14, 19], or even delaying care until they have returned to Canada [20]. These strategies leave a gap when it comes to planning for accessing timely preventative and diagnostic care while in the US, both of which are critical for reducing STI spread. Existing research in Florida indicates low STI testing rates among Canadian international retirement migrants while in the US despite engagement in high-risk behaviours [21].

The state of Arizona is a major US destination for Canadian retirement migrants due to infrastructure aimed at attracting seasonal and permanent-stay retirees [22, 23]. Yuma is an isolated, small city in the southwest part of Arizona that attracts sizeable numbers of both Canadian and American retirement migrants due to its warm, dry climate. The city offers numerous amenities for seniors, including recreational vehicle (RVs) parks, resorts, golf courses, and recreation centers [24, 25]. A 2018 study reported 71,000 seasonal winter visitors in Yuma County, contributing US$179 million in direct spending [26]. The deserts surrounding the city are popular for ‘boondocking,’ where retirees live temporarily in RVs on undeveloped land at little to no cost [27]. In addition, close proximity to Los Algodones, Mexico, allows easy access to affordable pharmaceuticals and dental care for visiting seniors [28]. Focus groups with health care providers in Yuma’s main hospital, the Yuma Regional Medical Center, highlighted concerns about risky sexual behaviours and inadequate safe sex education among visiting retirees, leading to what some thought were increased STI rates [8]. Although these rates are not tracked by local public health, popular news and social media pieces underscore concerns that risky sexual health practices and the spread of STIs are indeed happening in US retirement migrant communities [e.g., 29–31].

Addressing STI spread among older adults is highly stigmatized, often leading to a lack of discussion by both health care providers and patients [32–34]. This stigma extends to public health educational campaigns, which frequently overlook older adults, reinforcing the false perception that they are at lower risk for STIs [35–37]. Research evidence is needed to support countering such stigma and creating public health and health system interventions that are responsive to the needs of older adults [32], including international retirement migrants. Building from this recognition, in the current study we qualitatively explore STI transmission risks for Canadians wintering in Yuma. Drawing on key informant interviews with health care providers and senior administrators who have collectively supported care for hundreds of Canadian patients, we identify three types of risk that are challenged by the transnational nature of this seasonal travel practice. We draw on the findings to identify proactive, age-sensitive strategies for promoting sexual health and lessening STI spread.

## Methods

Our team of social science health researchers has been deeply exploring Canadian international retirement migrants’ access to health care while abroad [38–40]. Building on our prior research that has established Yuma as a popular destination for older Canadians [38, 41], in the current study we aimed to understand if this transnational practice introduced risks around contracting and/or treating STIs. We employed case study methodology, which focuses on understanding experiences within the context(s) in which they occur [42]. This methodological approach was chosen for its suitability in understanding complex social phenomena and building on existing relationships (i.e., years of research in Yuma involving local collaborators).

This study relied on interviewing key informants who could speak to relevant domains of expertise and had interacted with significant numbers of older Canadians. In January 2020, members of our team met with a collaborator at the Yuma Regional Medical Centre to identify the range of professional perspectives that may contribute expertise insight to the case study. Recognizing it was strategic to identify more professional groups to recruit from than the target number of participants, to account for position vacancies and recruitment challenges, 15 groups were identified with the goal of conducting at least ten interviews. To ensure breadth, it was agreed that no more than two representatives from a single professional group would be interviewed. Next, a semi-structured interview guide was collaboratively created using an iterative process of feedback and input among team members and the local collaborator. Ethics approval was then received from the Office of Research Ethics at Simon Fraser University.

Our collaborator at the Yuma Regional Medical Center identified potential participants. A team member reached out via email, inviting them to take part in a remote Zoom interview. This recruitment strategy led to six interviews being completed. The remaining four interviews were scheduled based on contacts identified through organizational websites, LinkedIn profiles, or introductions facilitated by existing interviewees. Outreach and interviewing took place from February 2023 to February 2024. This length was necessary to accommodate potential participants’ professional schedules and the seasonal nature of international retirement migration that placed exceptional demand on public health and health care systems for six months each year.

One-hour interviews were led by the second author, who had extensive on-site experience in Yuma and therefore familiarity with the local health care system and residential communities popular among older Canadians. The first author served as a note-taker. After obtaining verbal consent, the initial five questions contextualized professional roles and familiarity with international retirement migrants and STIs among older populations. The next questions probed: exposure to STIs and sexual behaviours (*n* = 4); access to care (*n* = 5); and preventative outreach opportunities (*n* = 3). To close, participants were asked if they wanted to touch on any additional matters or revisit any points they had raised. Following each interview, the interviewer and note-taker conducted a debrief recorded in fieldnotes that synthesized points of convergence with, and divergence from, other participants.

All interviews were digitally recorded and transcribed verbatim. Thematic analysis, which involves identifying and reporting patterns within qualitative datasets along with describing them in rich detail [43, 44], was undertaken. Our first analytic step involved two research team members independently reviewing transcripts to inductively and deductively identify conversational directions of significance. This led to the identification of two meta-themes for deeper analysis, one of which is explored herein. The scope and scale of the meta-themes were reviewed by the other team members to confirm dependability via investigator triangulation. The first author developed a coding scheme that organized themes and sub-themes following line-by-line transcript review. Team members provided feedback on the scheme, after which the transcripts were imported into NVivo in preparation for coding. Coded data extracts were shared to facilitate feedback on the scheme and its application, which was followed by a second round of coding. Upon finalization, extracts were again circulated to the team to support interpretation. Consistent with thematic analysis [45], the themes identified were contrasted against relevant existing literature and with our collaborator at Yuma Regional Medical Center. This process supported both the identification of novel findings and our engagement with the data. In the section that follows, we integrate verbatim quotes to support authenticity.

## Results

We interviewed key informant medical and public health practitioners as well as senior administrators based in Yuma (*n* = 10) who represented seven distinct professions. To lessen threats to anonymity given Yuma’s small size, we attribute verbatim quotes either to ‘health care providers’ or ‘senior administrators.’ On average, participants worked in Yuma’s health system for almost 13 years (x̅=12.95), which gave them considerable insight into factors such as seasonal patient influxes, health care use and demand among older Canadians, and generally the integration of these seasonal residents into the local landscape. While all participants were knowledgeable about medical care for retirement migrants, having interacted with or supported care for hundreds of Canadian international retirement migrants, their expertise in treating and/or preventing sexually transmitted diseases was meaningfully varied.

In its broadest sense, risk is conceptualized as the likelihood that being exposed to something hazardous will result in a negative outcome [46]. As noted above, older people oftentimes do not see themselves at significant risk of contracting STIs [36, 37]. Key informants did not concur with this view and raised three types of risks that could contribute to infection transmission. First, there were activities and practices inherent in the social environments associated with international retirement migration that had the potential to lead to exposure. Second, structural and personal factors posed barriers to accessing testing services and threatened diagnosis, thereby allowing continued transmission. And third, there were challenges associated with older Canadians obtaining treatment for diagnosed infections that could result in continued infection spread to others, or re-infection among those already diagnosed. We expand on these risks below, discussing each separately to deeply explore the sexual health landscape of international retirement migrants in Yuma as it relates to STIs.

### Risky activities

Participants depicted retirement migrant communities as heavily social, with RV parks serving as hubs for constant activity. This environment, described by participants as “*100% social*,” facilitated close interaction. A participant explained that “*there’s constantly stuff going on*,* events and all sorts of stuff. (…) It’s a whole different environment than [what] you might see when they’re in their regular places.*” The geographic proximity and continuous social gatherings contributed to an atmosphere where sexual health practices, including the use of prophylactics, may have been more relaxed due to a sense of trust among familiar residents. This presumption of safety, as one health care provider articulated, led to a diminished perception of risk: “*Maybe they feel like they can’t get any sexually transmitted disease*,* maybe they feel safer because it is like-minded individuals (…) the assumption is like*,* ‘Oh*,* they’re clean*,* we’re good’.*” This was echoed by a senior administrator who highlighted how international retirement migrants may have ruled out risk of pregnancy as a driver for using condoms but did not consider “*other health risks that are associated with having unprotected sex.*” Several participants agreed that such decisions were likely underpinned by generational differences in attitudes towards protection use during intimate encounters.

Lifestyle factors, notably drug (both recreational and prescribed) and alcohol consumption were discussed as contributing to increased sexual activity and, consequently, exposure to STIs. A health care provider noted that “*[cannabis] gummies [are] legal in Arizona now*” and highlighted the availability of sexual enhancement medications (e.g., Viagra, which could be easily accessed without prescription nearby in Mexico). A senior administrator noted that: “*We’re all very much aware of how easy it is to get that sort of stuff. (…) You just go across the border.*” Participants also noted that ‘boondockers’ who lived in make-shift RV encampments in the deserts surrounding Yuma and RV parks with the presence of “*swinger culture*” (i.e., couples engaging in intimate practices with other couples) presented particular exposure and transmission risks. Participants used terms such as “*big sex-capade*” to talk about these types of communities. They also referenced “*casserole widows*” who provided support in times of loss to illustrate the complex social fabric and opportunities for “*promiscuity within… communities [of] older adults.*” Several participants explained how this complexity was compounded by the presence of other mobile populations in Yuma, such as military personnel and migrant workers arriving from Mexico, that could lead to opportunities for transient sexual encounters. A health care provider with expertise related to disease transmission noted that this intersection of mobilities contributed to “*anecdotally higher*” STI rates in Yuma.

### Risky care access

Participants highlighted that there was likely underdiagnosis of STIs within international retirement migrant communities. A noted challenge was the known low adherence to testing and preventative measures among older Canadians while in Yuma, driven by a preference to receive care “*in their home country.*” This was often due to the prohibitive costs associated with screening services in the US, as they were typically not covered by the travel health emergency insurance plans available to older Canadians while in the US. A senior administrator explained that these seasonal visitors typically did not want to access care in Yuma, including diagnostic testing, if “*their insurance doesn’t pay for it.*” In more extreme cases, such as those residing in the isolated desert areas, the extensive distances they had to travel to seek health care and its associated costs served as a barrier to care access. According to participants, this added financial strain often led them to decline follow-up appointments, such as for testing, that required them to return to clinics in Yuma.

Participants, especially those in care provision settings, mentioned that it was common for providers to factor in uninsured costs when referring seniors for diagnostic tests due to the likelihood of pension reliance and/or limited budgets. It was acknowledged by participants that providers limited STI testing to avoid placing financial burden on older seasonal patients. Moreover, STIs were not typically considered as the first prognosis for older patients unless noted sexual activity precipitated symptoms, which further challenged diagnosis. A senior administrator remarked: “*Stigma. Yes*,* definitely. I’m sure that plays a part. But if you’re not having horrible symptoms*,* you probably wait till you get back home [to Canada] …because it’s not easy also getting a provider here.*” The reluctance to engage in conversations about sexual history, combined with the absence of established local primary care relationships for Canadians visiting seasonally, were commonly identified as critical barriers to the timely detection of STIs. Such factors highlighted the complex interplay of individual and system-based challenges that may prevent access to diagnostic care.

### Risky treatment scenarios

Treatment of STIs contracted by Canadian international retirement migrants in Yuma was fraught with challenges. Participants expressed concerns surrounding the risks of reinfection due to continued transmission within residential communities, even if some members had been diagnosed and treated, and a likely general underestimation of infection risks due to transmission misconceptions. This laidback attitude, coupled with the transient nature of the retirement migrant population, complicated treatment efforts. A health care provider asked: “*Are they going to recollect who they need to contact? Because you need to bring them in to get treatment. (…) What if you give them a pill and you never see them and they end up with… side effects?*” Even for Canadian international retirement migrants who were able to access diagnostic services through referral from primary care, participants noted that the seasonal nature of their time in Yuma disrupted standard treatment protocols. There were also barriers to ensuring care continuity upon return to Canada due to differences in medical record-keeping practices and a general lack of cross-border care coordination.

Further compounding treatment challenges were logistical issues, such as the lack of a permanent address in Yuma, which hampered efforts for providers to ensure timely treatment and follow-up care. A participant asked: “*…how are you going to reach them? Are they going to send a letter to their house? Are they going to go knock on their doors?*” Moreover, international retirement migrants had the option to receive treatment at a primary care clinic or Yuma County’s Sexually Transmitted Disease Clinic. Information exchange between these clinics was limited, which served as a systemic informational barrier that could lead to uncertainty regarding whether treatment was obtained. Several care providers pointed out the difficulty of reaching out to sexual partners about a potential transmission given the sensitivity of the subject: “*What if they’ve been with multiple partners and are embarrassed to go call them and tell them*,* ‘Hey*,* you need to get treated’.*” The sensitive nature of contacting sexual partners about a potential transmission and encouraging timely diagnosis and treatment, within the close-knit social context of retirement migrant communities, created a challenging context for ensuring access to treatment.

## Discussion

The findings reveal nuanced understandings of risk as it related to STI transmission among Canadian international retirement migrants wintering in Yuma, Arizona. Complex social dynamics, barriers to care, and inconsistent treatment adherence collectively contributed to what was perceived by some key informants as an elevated risk of STI exposure among older Canadians in this seasonal transnational travel context. The findings support calls for urgent targeted interventions, such as enhanced sexual health education, tailored to older adults [47]. They also underscore the need for more comprehensive travel health insurance coverage to be made available to Canadian international retirement migrants, given their long stays abroad that may necessitate access to preventative or diagnostic care. In the remainder of this discussion, we contextualize the findings within the existing literature, highlighting implications for those supporting the health of older Canadians wintering in Yuma.

This analysis confirms existing research documenting the social nature of international retirement migrant communities [48–50], which foster environments conducive to increased sexual interaction [51]. Key informants concurred that activities in these environments may also lead to increased exposure to STIs. This is consistent with travel health research that has shown that extended travel coupled with alcohol and drug consumption significantly escalates sexual risk-taking behaviours [1, 2, 4]. Challenges in health care access documented by the key informants, particularly in diagnostics – which are often compounded by systemic barriers and insurance limitations [11, 52, 53] – have also been identified elsewhere. Shown here, these challenges may hinder timely access to diagnosis and treatment for STIs. The stigma surrounding discussions of sexual histories with older patients identified by participants is also well established [32–34]. Stigma-indifferent care practices may inadvertently lessen access to diagnosis and treatment. Transmission risk is further amplified by the implicit trust that Canadian international retirement migrants have in their peers [37, 47] that, according to participants, may extend to considering them to be ‘safe’ sex partners. These are among the established factors that contribute to the documented increase in STIs among older people [18, 32, 36], some of which are amplified in the context of international retirement migration as identified by the key informants.

The critical role that primary care can play in diagnosing STIs and supporting treatment among Canadian international retirement migrants was underscored by participants. Previous research in Yuma shows that older Canadians, with their experience of residing there seasonally and common access to local information, are reasonably adept at navigating the local health care system for acute care needs or planned chronic illness management [8, 20]. However, the current analysis points to a lack of established, ongoing relationships with primary care physicians serving as a barrier when it comes to gaining access to STI diagnostics. This discrepancy highlights a critical gap between the available resources and the types of health care these long-stay travellers need to protect their sexual health, emphasizing the need for targeted interventions to bridge this divide. Addressing this gap may provide more opportunities to discuss sexual health and receive tailored advice, which is essential for managing health in a mobile lifestyle [6, 54]. Furthermore, the presence of a sexually promiscuous scene, akin to those reported in other retirement migrant communities in the US [55, 56], was noted to be present in Yuma. This observation compels a re-evaluation of common perceptions about aging and sexual activity that address the pervasive stigma surrounding sexual health discussions in older populations through targeted training for public health and primary care providers in Yuma and other popular destinations for international retirement migrants, thus improving STI reporting and prevention.

These findings hold critical implications for public health interventions and health care practice, calling attention to the necessity for comprehensive, culturally sensitive, and age-appropriate approaches to improve sexual health among Canadian international retirement migrants in the US. Routine screening initiatives are crucial to destigmatizing STIs [57] and promoting open discussions about sexual health, which should also include distributing informational materials [58]. Popular destinations like Yuma should explore if and how existing screening initiatives can be extended to include Canadian international retirement migrants given their sizeable seasonal presence. Efforts to normalize testing for STIs, such as by offering home-based sample collection kits [59] – which may enhance testing accessibility for long-stay travellers – are critical. Information on safer sex practices should also be amplified through targeted public health campaigns [33]. To further enhance these initiatives, integrating telehealth services may support better care continuity given the transnational context of international retirement migration and the fact that diagnosis may happen in a different jurisdiction than treatment [59, 60]. The effectiveness of travel medicine consultations must also be leveraged to provide pre- and post-travel sexual health education, which has been shown to reduce risky behaviours and STI rates among international travellers [6, 54].

To deepen understandings of STIs and care provision among international retirement migrants, future research should focus on developing intervention models that effectively reduce stigma. Universal screening programs are a promising area, with the potential to transform perceptions and behaviours towards STIs across cultures and care systems [57, 61]. Further investigation into community-based initiatives and partnerships with local organizations can provide valuable insights into how sexual health literacy and risk communication can be enhanced across diverse retirement destinations. Longitudinal studies are also needed to assess the impact of policy or process-driven changes on care access and outcomes. Such studies can help to pinpoint areas where assistance to facilitate care access is most needed [62, 63]. Expanding research to include other migratory populations and geographic contexts would support comparative analysis and enhance developing a more global understanding of the health challenges faced by retirement migrants across varied social contexts. Such comprehensive research efforts are essential for developing inclusive health interventions for these transnational travellers.

### Strengths & limitations

One of the major strengths of this case study is the collaboration with an on-site partner at the Yuma Regional Medical Center. Their involvement throughout ensured the findings are relevant to knowledge user communities. One key strength is that we interviewed a range of key informants from Yuma’s public health and health care sectors. Despite their differently situated professional backgrounds, we were able to identify consistent approaches to understanding risk. Additionally, we used triangulated processes throughout data analysis to support building rigour and credibility in the analytic process [64]. Investigator triangulation specifically assisted with determining the scope and scale of each risk theme, thus supporting the reliability of interpretation. An important limitation was our use of remote interviews. While offering logistical flexibility, they limited capturing non-verbal cues, which are often crucial for understanding the nuances of complex social interactions [65]. Nevertheless, it was our use of this medium that enabled data collection to take place and so, this consideration outweighed any concerns about not fully capturing non-verbal language.

## Conclusion

This study has provided significant new insights into the sexual health risks encountered by Canadian international retirement migrants wintering in Yuma, Arizona. Through in-depth interviews with key informant health practitioners and senior administrators, we have highlighted the complex dynamics that increase these older travellers’ susceptibility to STI transmission. Specifically, we identified three principal areas of risk: social practices within retirement migrant communities that increase exposure, barriers to accessing diagnostic services, and challenges in obtaining effective treatment. The transient nature of older Canadians’ seasonal stays in the US exacerbates these risks, disrupting continuity of care and emphasizing the urgent need for targeted public health interventions, tailored to the unique circumstances of older adults in international retirement settings. Training public health and primary care providers in destination communities to proactively discuss sexual health and tackle stigma may help navigate the complexities of ageing and sexual activity, positively impacting STI transmission among older Canadians wintering in the US.

## Data Availability

To maintain participants’ privacy and anonymity, interview transcripts from this study are not publicly available.

## Abbreviations

RV: Recreational vehicle
STI: Sexually transmitted infection
US: United States
